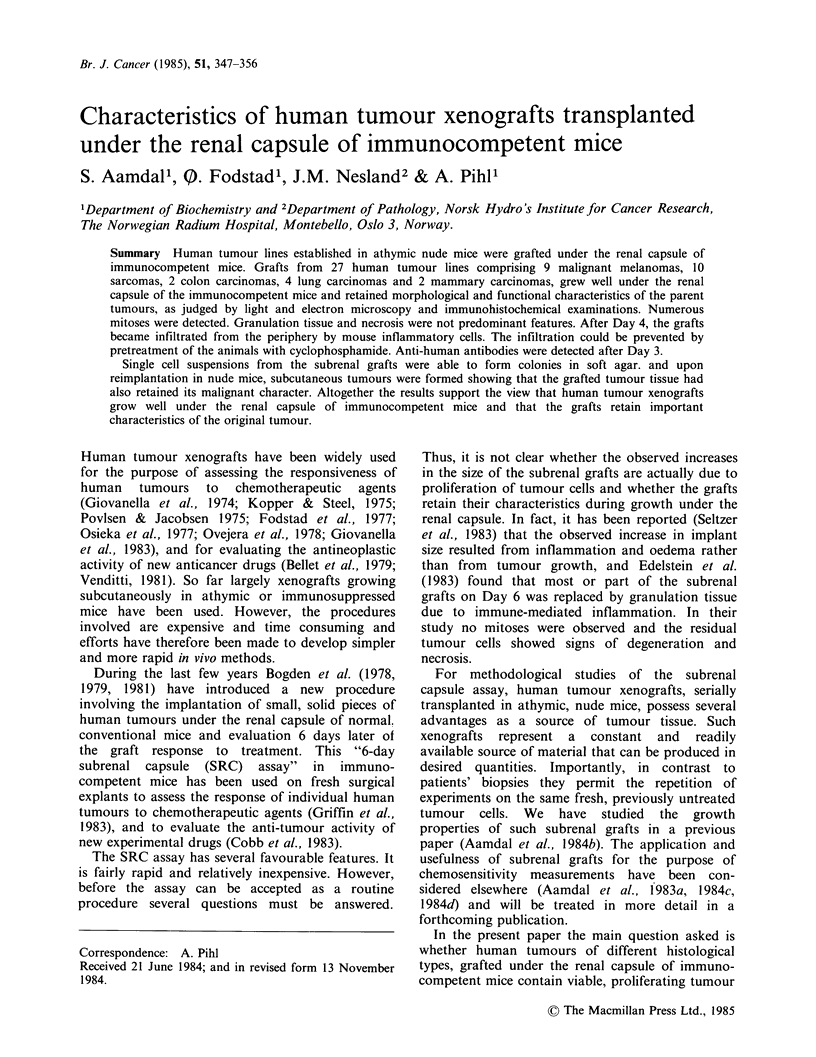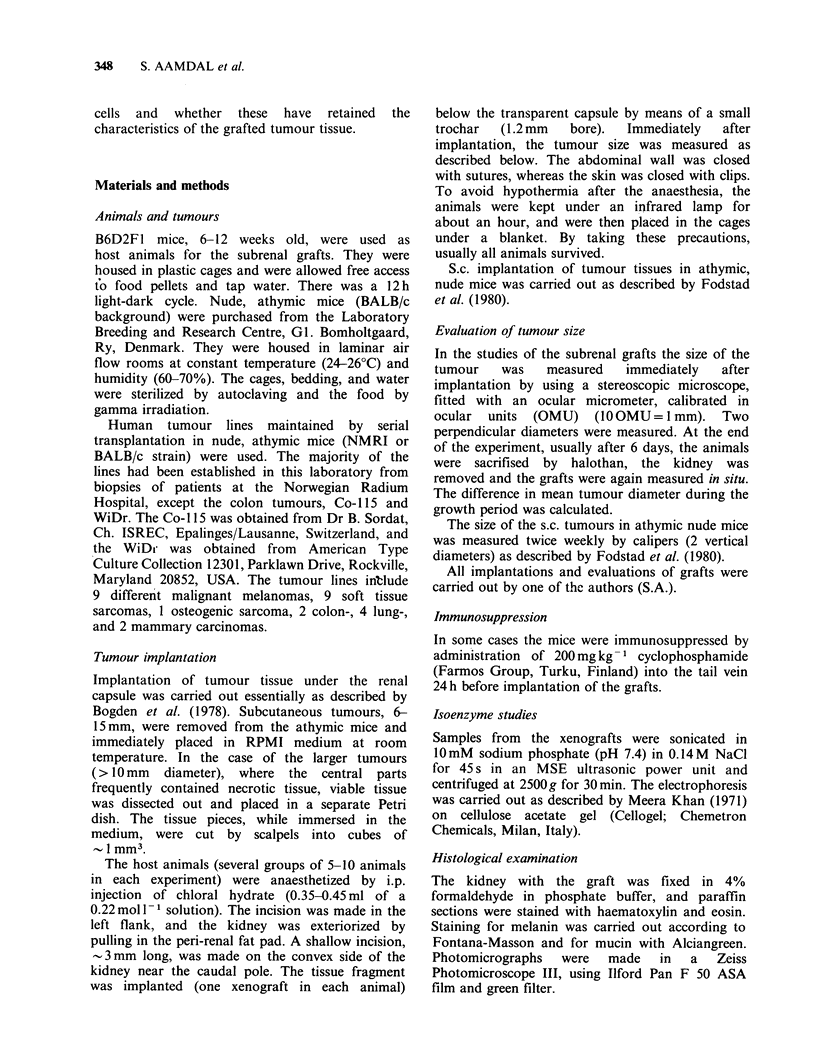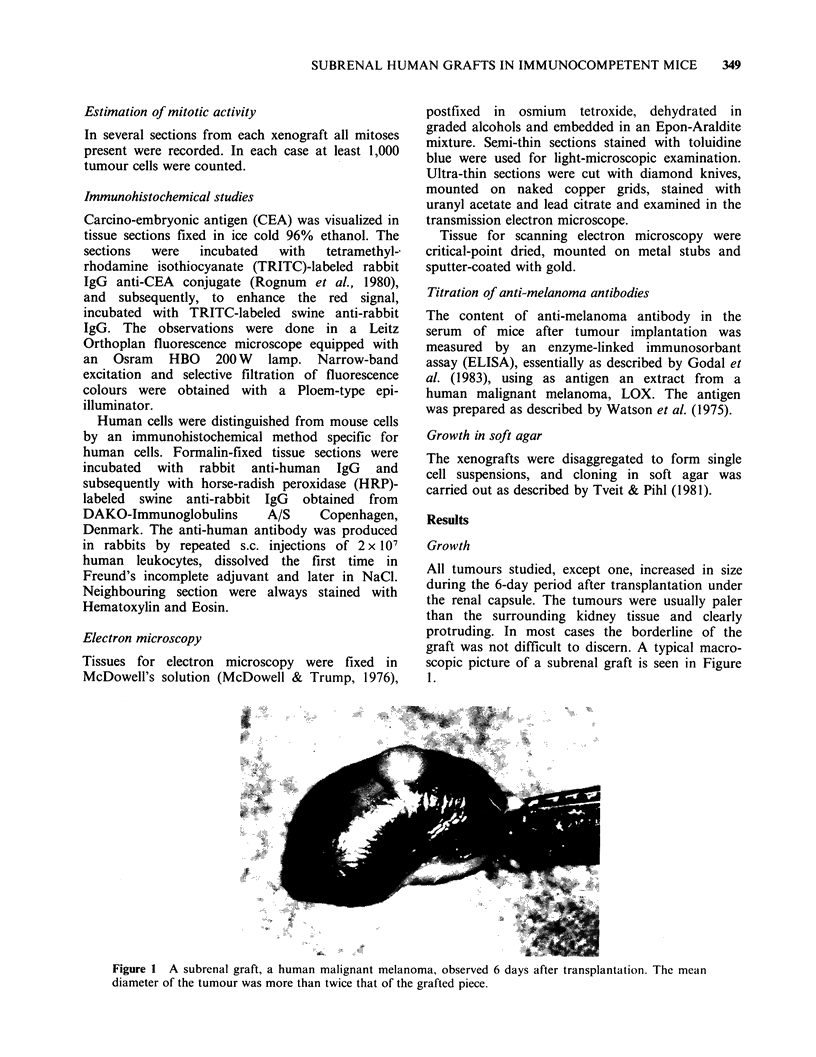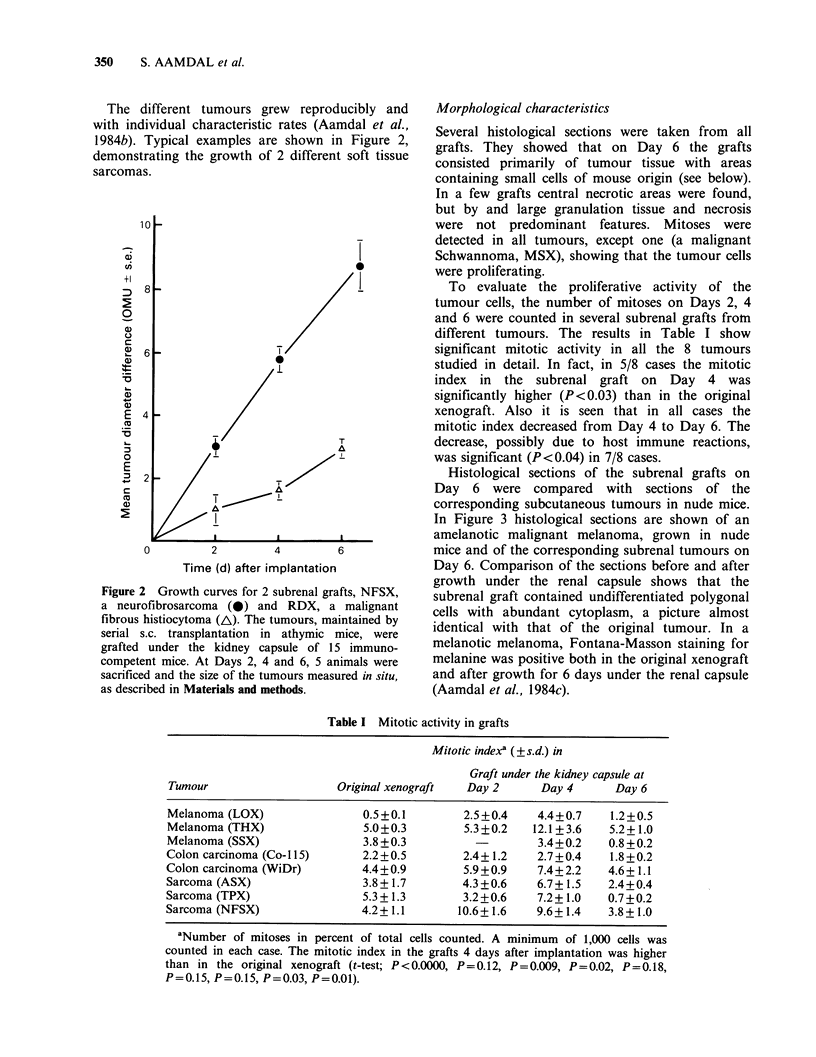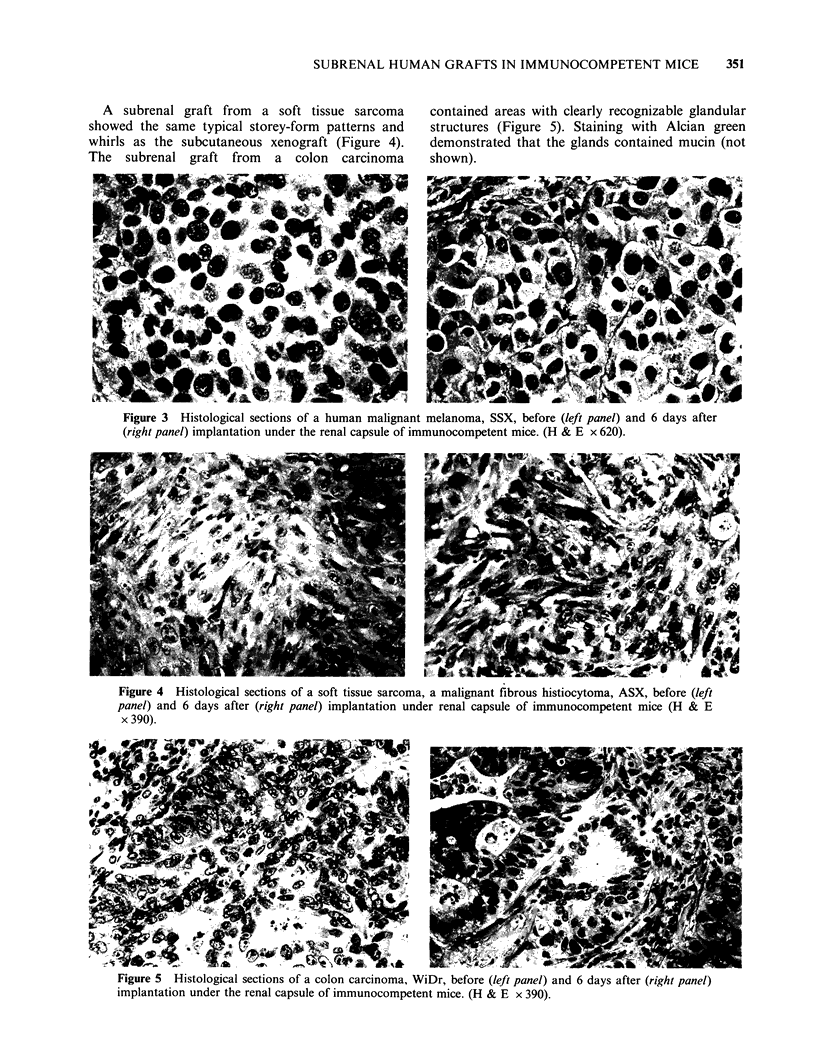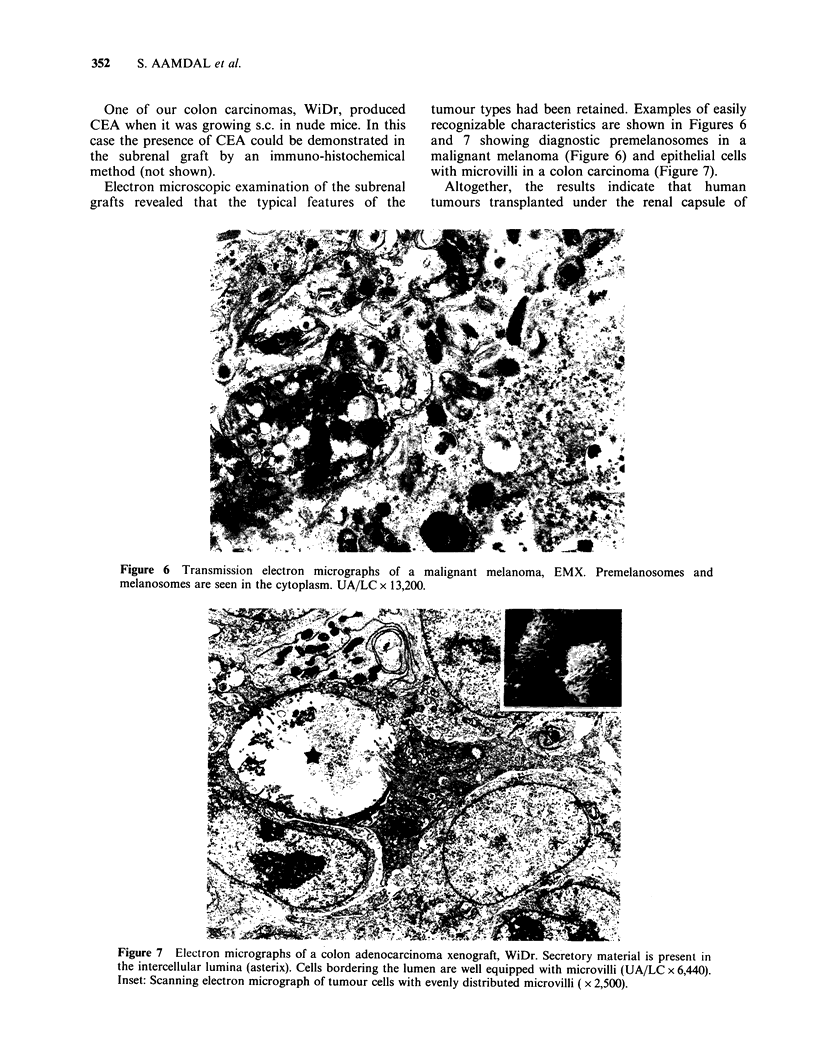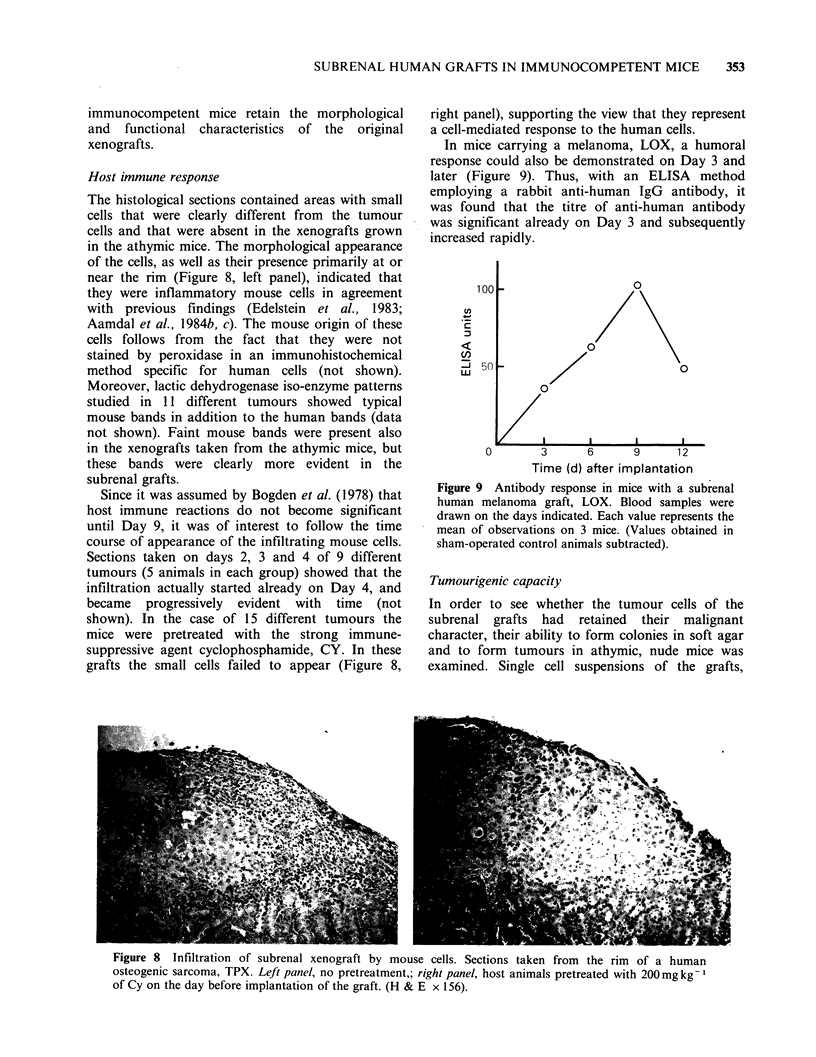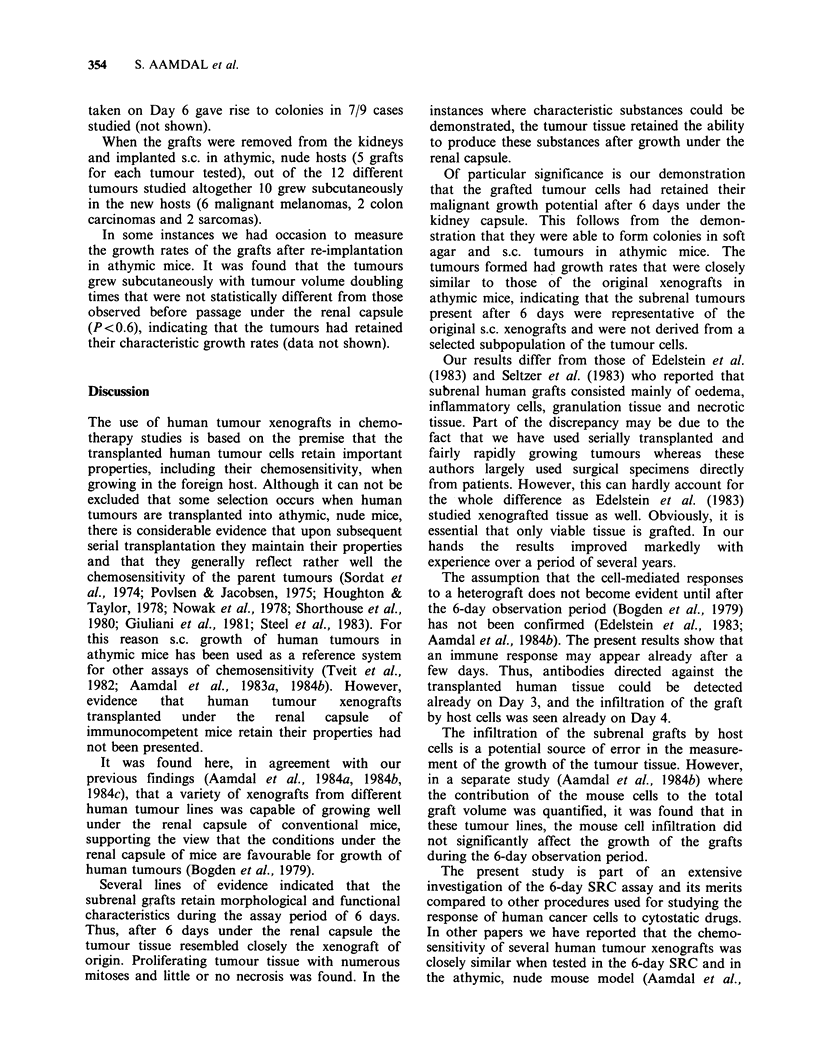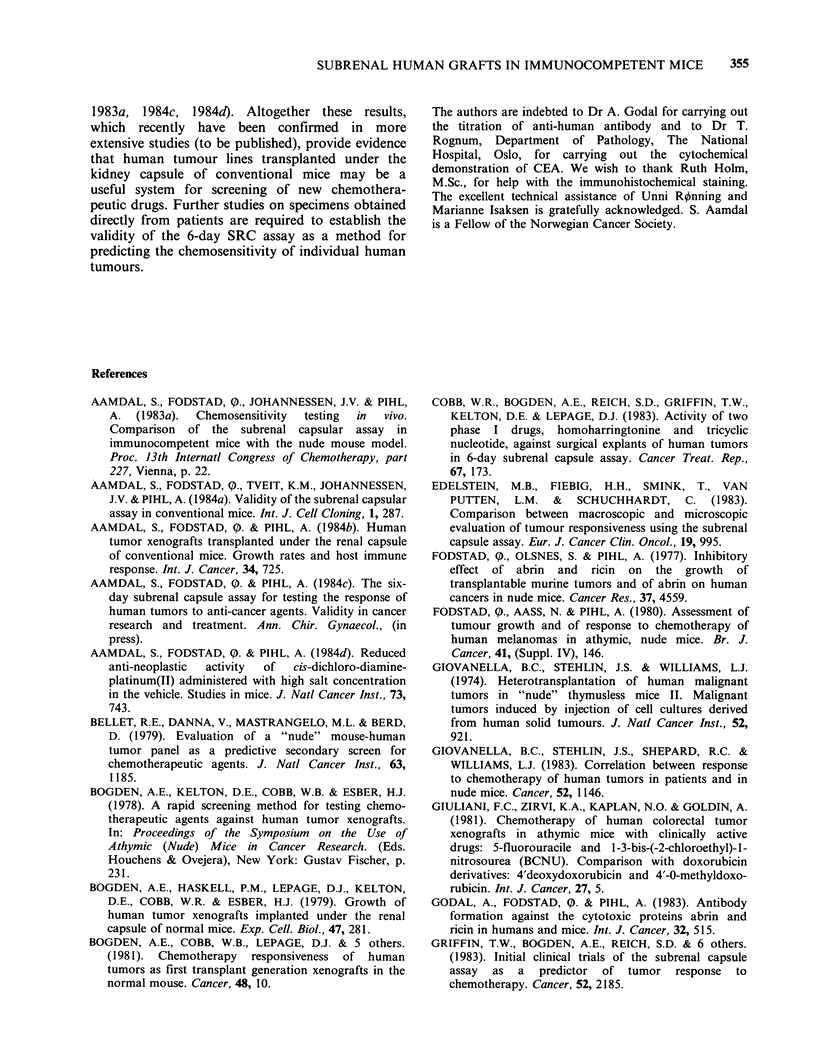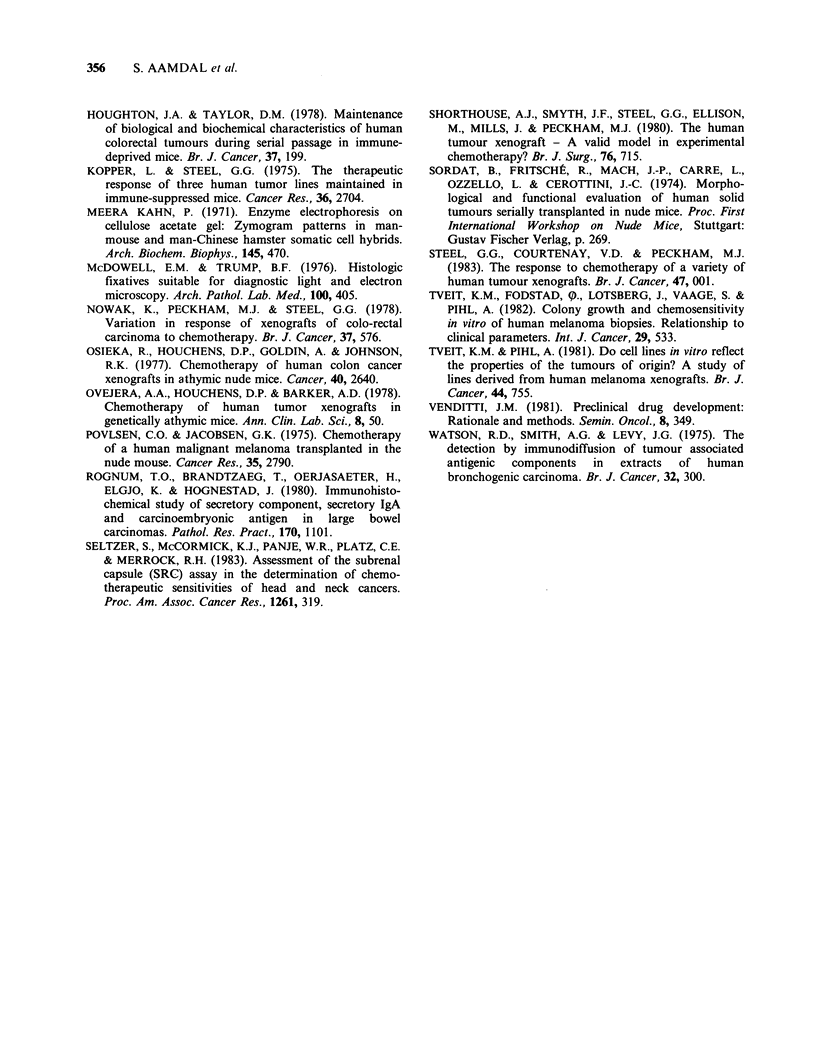# Characteristics of human tumour xenografts transplanted under the renal capsule of immunocompetent mice.

**DOI:** 10.1038/bjc.1985.46

**Published:** 1985-03

**Authors:** S. Aamdal, O. Fodstad, J. M. Nesland, A. Pihl

## Abstract

**Images:**


*Br. J. Cancer* (1985), **51**, 347–356

### Characteristics of human tumour xenografts transplanted under the renal capsule of immunocompetent mice

S. Aamdal[1], Ø. Fodstad[1], J.M. Nesland[2] & A. Pihl[1]

[1]*Department of Biochemistry and* [2]*Department of Pathology, Norsk Hydro's Institute for Cancer Research, The Norwegian Radium Hospital, Montebello, Oslo 3, Norway.*

**Summary** Human tumour lines established in athymic nude mice were grafted under the renal capsule of immunocompetent mice. Grafts from 27 human tumour lines comprising 9 malignant melanomas, 10 sarcomas, 2 colon carcinomas, 4 lung carcinomas and 2 mammary carcinomas, grew well under the renal capsule of the immunocompetent mice and retained morphological and functional characteristics of the parent tumours, as judged by light and electron microscopy and immunohistochemical examinations. Numerous mitoses were detected. Granulation tissue and necrosis were not predominant features. After Day 4, the grafts became infiltrated from the periphery by mouse inflammatory cells. The infiltration could be prevented by pretreatment of the animals with cyclophosphamide. Anti-human antibodies were detected after Day 3.

Single cell suspensions from the subrenal grafts were able to form colonies in soft agar. and upon reimplantation in nude mice, subcutaneous tumours were formed showing that the grafted tumour tissue had also retained its malignant character. Altogether the results support the view that human tumour xenografts grow well under the renal capsule of immunocompetent mice and that the grafts retain important characteristics of the original tumour.

Human tumour xenografts have been widely used for the purpose of assessing the responsiveness of human tumours to chemotherapeutic agents (Giovanella *et al.,* 1974; Kopper & Steel, 1975; Povlsen & Jacobsen 1975; Fodstad *et al.,* 1977; Osieka *et al.,* 1977; Ovejera *et al.,* 1978; Giovanella *et al.,* 1983), and for evaluating the antineoplastic activity of new anticancer drugs (Bellet *et al.,* 1979; Venditti, 1981). So far largely xenografts growing subcutaneously in athymic or immunosuppressed mice have been used. However, the procedures involved are expensive and time consuming and efforts have therefore been made to develop simpler and more rapid *in vivo* methods.

During the last few years Bogden *et al.* (1978, 1979, 1981) have introduced a new procedure involving the implantation of small, solid pieces of human tumours under the renal capsule of normal, conventional mice and evaluation 6 days later of the graft response to treatment. This "6-day subrenal capsule (SRC) assay" in immunocompetent mice has been used on fresh surgical explants to assess the response of individual human tumours to chemotherapeutic agents (Griffin *et al.,* 1983), and to evaluate the anti-tumour activity of new experimental drugs (Cobb *et al.,* 1983).

The SRC assay has several favourable features. It is fairly rapid and relatively inexpensive. However, before the assay can be accepted as a routine procedure several questions must be answered. Thus, it is not clear whether the observed increases in the size of the subrenal grafts are actually due to proliferation of tumour cells and whether the grafts retain their characteristics during growth under the renal capsule. In fact, it has been reported (Seltzer *et al.,* 1983) that the observed increase in implant size resulted from inflammation and oedema rather than from tumour growth, and Edelstein *et al.* (1983) found that most or part of the subrenal grafts on Day 6 was replaced by granulation tissue due to immune-mediated inflammation. In their study no mitoses were observed and the residual tumour cells showed signs of degeneration and necrosis.

For methodological studies of the subrenal capsule assay, human tumour xenografts, serially transplanted in athymic, nude mice, possess several advantages as a source of tumour tissue. Such xenografts represent a constant and readily available source of material that can be produced in desired quantities. Importantly, in contrast to patients' biopsies they permit the repetition of experiments on the same fresh, previously untreated tumour cells. We have studied the growth properties of such subrenal grafts in a previous paper (Aamdal *et al.,* 1984*b*). The application and usefulness of subrenal grafts for the purpose of chemosensitivity measurements have been considered elsewhere (Aamdal *et al.,* 1983*a*, 1984*c*, 1984*d*) and will be treated in more detail in a forthcoming publication.

In the present paper the main question asked is whether human tumours of different histological types, grafted under the renal capsule of immunocompetent mice contain viable, proliferating tumour

Correspondence: A. Pihl
Received 21 June 1984; and in revised form 13 November 1984.

© The Macmillan Press Ltd., 1985

cells and whether these have retained the characteristics of the grafted tumour tissue.

### Materials and methods

### *Animals and tumours*

B6D2F1 mice, 6–12 weeks old, were used as host animals for the subrenal grafts. They were housed in plastic cages and were allowed free access to food pellets and tap water. There was a 12 h light-dark cycle. Nude, athymic mice (BALB/c background) were purchased from the Laboratory Breeding and Research Centre, G1. Bomholtgaard, Ry, Denmark. They were housed in laminar air flow rooms at constant temperature (24–26°C) and humidity (60–70%). The cages, bedding, and water were sterilized by autoclaving and the food by gamma irradiation.

Human tumour lines maintained by serial transplantation in nude, athymic mice (NMRI or BALB/c strain) were used. The majority of the lines had been established in this laboratory from biopsies of patients at the Norwegian Radium Hospital, except the colon tumours, Co-115 and WiDr. The Co-115 was obtained from Dr B. Sordat, Ch. ISREC, Epalinges/Lausanne, Switzerland, and the WiDr was obtained from American Type Culture Collection 12301, Parklawn Drive, Rockville, Maryland 20852, USA. The tumour lines include 9 different malignant melanomas, 9 soft tissue sarcomas, 1 osteogenic sarcoma, 2 colon-, 4 lung-, and 2 mammary carcinomas.

### *Tumour implantation*

Implantation of tumour tissue under the renal capsule was carried out essentially as described by Bogden *et al.* (1978). Subcutaneous tumours, 6–15 mm, were removed from the athymic mice and immediately placed in RPMI medium at room temperature. In the case of the larger tumours (>10 mm diameter), where the central parts frequently contained necrotic tissue, viable tissue was dissected out and placed in a separate Petri dish. The tissue pieces, while immersed in the medium, were cut by scalpels into cubes of $\sim 1\ mm^3$.

The host animals (several groups of 5–10 animals in each experiment) were anaesthetized by i.p. injection of chloral hydrate (0.35–0.45 ml of a $0.22\ mol\ l^{-1}$ solution). The incision was made in the left flank, and the kidney was exteriorized by pulling in the peri-renal fat pad. A shallow incision, ~3 mm long, was made on the convex side of the kidney near the caudal pole. The tissue fragment was implanted (one xenograft in each animal) below the transparent capsule by means of a small trochar (1.2 mm bore). Immediately after implantation, the tumour size was measured as described below. The abdominal wall was closed with sutures, whereas the skin was closed with clips. To avoid hypothermia after the anaesthesia, the animals were kept under an infrared lamp for about an hour, and were then placed in the cages under a blanket. By taking these precautions, usually all animals survived.

S.c. implantation of tumour tissues in athymic, nude mice was carried out as described by Fodstad *et al.* (1980).

### *Evaluation of tumour size*

In the studies of the subrenal grafts the size of the tumour was measured immediately after implantation by using a stereoscopic microscope, fitted with an ocular micrometer, calibrated in ocular units (OMU) (10 OMU = 1 mm). Two perpendicular diameters were measured. At the end of the experiment, usually after 6 days, the animals were sacrifised by halothan, the kidney was removed and the grafts were again measured *in situ*. The difference in mean tumour diameter during the growth period was calculated.

The size of the s.c. tumours in athymic nude mice was measured twice weekly by calipers (2 vertical diameters) as described by Fodstad *et al.* (1980).

All implantations and evaluations of grafts were carried out by one of the authors (S.A.).

### *Immunosuppression*

In some cases the mice were immunosuppressed by administration of $200\ mg\ kg^{-1}$ cyclophosphamide (Farmos Group, Turku, Finland) into the tail vein 24 h before implantation of the grafts.

### *Isoenzyme studies*

Samples from the xenografts were sonicated in 10 mM sodium phosphate (pH 7.4) in 0.14 M NaCl for 45 s in an MSE ultrasonic power unit and centrifuged at 2500 *g* for 30 min. The electrophoresis was carried out as described by Meera Khan (1971) on cellulose acetate gel (Cellogel; Chemetron Chemicals, Milan, Italy).

### *Histological examination*

The kidney with the graft was fixed in 4% formaldehyde in phosphate buffer, and paraffin sections were stained with haematoxylin and eosin. Staining for melanin was carried out according to Fontana-Masson and for mucin with Alciangreen. Photomicrographs were made in a Zeiss Photomicroscope III, using Ilford Pan F 50 ASA film and green filter.

*Estimation of mitotic activity*

In several sections from each xenograft all mitoses present were recorded. In each case at least 1,000 tumour cells were counted.

*Immunohistochemical studies*

Carcino-embryonic antigen (CEA) was visualized in tissue sections fixed in ice cold 96% ethanol. The sections were incubated with tetramethyl-rhodamine isothiocyanate (TRITC)-labeled rabbit IgG anti-CEA conjugate (Rognum *et al.*, 1980), and subsequently, to enhance the red signal, incubated with TRITC-labeled swine anti-rabbit IgG. The observations were done in a Leitz Orthoplan fluorescence microscope equipped with an Osram HBO 200 W lamp. Narrow-band excitation and selective filtration of fluorescence colours were obtained with a Ploem-type epi-illuminator.

Human cells were distinguished from mouse cells by an immunohistochemical method specific for human cells. Formalin-fixed tissue sections were incubated with rabbit anti-human IgG and subsequently with horse-radish peroxidase (HRP)-labeled swine anti-rabbit IgG obtained from DAKO-Immunoglobulins A/S Copenhagen, Denmark. The anti-human antibody was produced in rabbits by repeated s.c. injections of $2 \times 10^7$ human leukocytes, dissolved the first time in Freund's incomplete adjuvant and later in NaCl. Neighbouring section were always stained with Hematoxylin and Eosin.

*Electron microscopy*

Tissues for electron microscopy were fixed in McDowell's solution (McDowell & Trump, 1976), postfixed in osmium tetroxide, dehydrated in graded alcohols and embedded in an Epon-Araldite mixture. Semi-thin sections stained with toluidine blue were used for light-microscopic examination. Ultra-thin sections were cut with diamond knives, mounted on naked copper grids, stained with uranyl acetate and lead citrate and examined in the transmission electron microscope.

Tissue for scanning electron microscopy were critical-point dried, mounted on metal stubs and sputter-coated with gold.

*Titration of anti-melanoma antibodies*

The content of anti-melanoma antibody in the serum of mice after tumour implantation was measured by an enzyme-linked immunosorbant assay (ELISA), essentially as described by Godal *et al.* (1983), using as antigen an extract from a human malignant melanoma, LOX. The antigen was prepared as described by Watson *et al.* (1975).

*Growth in soft agar*

The xenografts were disaggregated to form single cell suspensions, and cloning in soft agar was carried out as described by Tveit & Pihl (1981).

### Results

*Growth*

All tumours studied, except one, increased in size during the 6-day period after transplantation under the renal capsule. The tumours were usually paler than the surrounding kidney tissue and clearly protruding. In most cases the borderline of the graft was not difficult to discern. A typical macroscopic picture of a subrenal graft is seen in Figure 1.

**Figure 1** A subrenal graft, a human malignant melanoma, observed 6 days after transplantation. The mean diameter of the tumour was more than twice that of the grafted piece.

The different tumours grew reproducibly and with individual characteristic rates (Aamdal *et al.*, 1984*b*). Typical examples are shown in Figure 2, demonstrating the growth of 2 different soft tissue sarcomas.

**Figure 2** Growth curves for 2 subrenal grafts, NFSX, a neurofibrosarcoma (●) and RDX, a malignant fibrous histiocytoma (△). The tumours, maintained by serial s.c. transplantation in athymic mice, were grafted under the kidney capsule of 15 immunocompetent mice. At Days 2, 4 and 6, 5 animals were sacrificed and the size of the tumours measured *in situ*, as described in **Materials and methods**.

*Morphological characteristics*

Several histological sections were taken from all grafts. They showed that on Day 6 the grafts consisted primarily of tumour tissue with areas containing small cells of mouse origin (see below). In a few grafts central necrotic areas were found, but by and large granulation tissue and necrosis were not predominant features. Mitoses were detected in all tumours, except one (a malignant Schwannoma, MSX), showing that the tumour cells were proliferating.

To evaluate the proliferative activity of the tumour cells, the number of mitoses on Days 2, 4 and 6 were counted in several subrenal grafts from different tumours. The results in Table I show significant mitotic activity in all the 8 tumours studied in detail. In fact, in 5/8 cases the mitotic index in the subrenal graft on Day 4 was significantly higher ($P<0.03$) than in the original xenograft. Also it is seen that in all cases the mitotic index decreased from Day 4 to Day 6. The decrease, possibly due to host immune reactions, was significant ($P<0.04$) in 7/8 cases.

Histological sections of the subrenal grafts on Day 6 were compared with sections of the corresponding subcutaneous tumours in nude mice. In Figure 3 histological sections are shown of an amelanotic malignant melanoma, grown in nude mice and of the corresponding subrenal tumours on Day 6. Comparison of the sections before and after growth under the renal capsule shows that the subrenal graft contained undifferentiated polygonal cells with abundant cytoplasm, a picture almost identical with that of the original tumour. In a melanotic melanoma, Fontana-Masson staining for melanine was positive both in the original xenograft and after growth for 6 days under the renal capsule (Aamdal *et al.*, 1984*c*).

**Table I** Mitotic activity in grafts

| | Mitotic index[a] (±*s.d.*) in | | | |
|---|---|---|---|---|
| | | Graft under the kidney capsule at | | |
| *Tumour* | *Original xenograft* | *Day 2* | *Day 4* | *Day 6* |
| Melanoma (LOX) | 0.5±0.1 | 2.5±0.4 | 4.4±0.7 | 1.2±0.5 |
| Melanoma (THX) | 5.0±0.3 | 5.3±0.2 | 12.1±3.6 | 5.2±1.0 |
| Melanoma (SSX) | 3.8±0.3 | — | 3.4±0.2 | 0.8±0.2 |
| Colon carcinoma (Co-115) | 2.2±0.5 | 2.4±1.2 | 2.7±0.4 | 1.8±0.2 |
| Colon carcinoma (WiDr) | 4.4±0.9 | 5.9±0.9 | 7.4±2.2 | 4.6±1.1 |
| Sarcoma (ASX) | 3.8±1.7 | 4.3±0.6 | 6.7±1.5 | 2.4±0.4 |
| Sarcoma (TPX) | 5.3±1.3 | 3.2±0.6 | 7.2±1.0 | 0.7±0.2 |
| Sarcoma (NFSX) | 4.2±1.1 | 10.6±1.6 | 9.6±1.4 | 3.8±1.0 |

[a]Number of mitoses in percent of total cells counted. A minimum of 1,000 cells was counted in each case. The mitotic index in the grafts 4 days after implantation was higher than in the original xenograft (*t*-test; $P<0.0000$, $P=0.12$, $P=0.009$, $P=0.02$, $P=0.18$, $P=0.15$, $P=0.15$, $P=0.03$, $P=0.01$).

A subrenal graft from a soft tissue sarcoma showed the same typical storey-form patterns and whirls as the subcutaneous xenograft (Figure 4). The subrenal graft from a colon carcinoma contained areas with clearly recognizable glandular structures (Figure 5). Staining with Alcian green demonstrated that the glands contained mucin (not shown).

**Figure 3** Histological sections of a human malignant melanoma, SSX, before (*left panel*) and 6 days after (*right panel*) implantation under the renal capsule of immunocompetent mice. (H & E ×620).

**Figure 4** Histological sections of a soft tissue sarcoma, a malignant fibrous histiocytoma, ASX, before (*left panel*) and 6 days after (*right panel*) implantation under renal capsule of immunocompetent mice (H & E ×390).

**Figure 5** Histological sections of a colon carcinoma, WiDr, before (*left panel*) and 6 days after (*right panel*) implantation under the renal capsule of immunocompetent mice. (H & E ×390).

One of our colon carcinomas, WiDr, produced CEA when it was growing s.c. in nude mice. In this case the presence of CEA could be demonstrated in the subrenal graft by an immuno-histochemical method (not shown).

Electron microscopic examination of the subrenal grafts revealed that the typical features of the tumour types had been retained. Examples of easily recognizable characteristics are shown in Figures 6 and 7 showing diagnostic premelanosomes in a malignant melanoma (Figure 6) and epithelial cells with microvilli in a colon carcinoma (Figure 7).

Altogether, the results indicate that human tumours transplanted under the renal capsule of

**Figure 6** Transmission electron micrographs of a malignant melanoma, EMX. Premelanosomes and melanosomes are seen in the cytoplasm. UA/LC × 13,200.

**Figure 7** Electron micrographs of a colon adenocarcinoma xenograft, WiDr. Secretory material is present in the intercellular lumina (asterix). Cells bordering the lumen are well equipped with microvilli (UA/LC × 6,440). Inset: Scanning electron micrograph of tumour cells with evenly distributed microvilli ( × 2,500).

immunocompetent mice retain the morphological and functional characteristics of the original xenografts.

*Host immune response*

The histological sections contained areas with small cells that were clearly different from the tumour cells and that were absent in the xenografts grown in the athymic mice. The morphological appearance of the cells, as well as their presence primarily at or near the rim (Figure 8, left panel), indicated that they were inflammatory mouse cells in agreement with previous findings (Edelstein *et al.*, 1983; Aamdal *et al.*, 1984*b*, *c*). The mouse origin of these cells follows from the fact that they were not stained by peroxidase in an immunohistochemical method specific for human cells (not shown). Moreover, lactic dehydrogenase iso-enzyme patterns studied in 11 different tumours showed typical mouse bands in addition to the human bands (data not shown). Faint mouse bands were present also in the xenografts taken from the athymic mice, but these bands were clearly more evident in the subrenal grafts.

Since it was assumed by Bogden *et al.* (1978) that host immune reactions do not become significant until Day 9, it was of interest to follow the time course of appearance of the infiltrating mouse cells. Sections taken on days 2, 3 and 4 of 9 different tumours (5 animals in each group) showed that the infiltration actually started already on Day 4, and became progressively evident with time (not shown). In the case of 15 different tumours the mice were pretreated with the strong immune-suppressive agent cyclophosphamide, CY. In these grafts the small cells failed to appear (Figure 8, right panel), supporting the view that they represent a cell-mediated response to the human cells.

In mice carrying a melanoma, LOX, a humoral response could also be demonstrated on Day 3 and later (Figure 9). Thus, with an ELISA method employing a rabbit anti-human IgG antibody, it was found that the titre of anti-human antibody was significant already on Day 3 and subsequently increased rapidly.

**Figure 9** Antibody response in mice with a subrenal human melanoma graft, LOX. Blood samples were drawn on the days indicated. Each value represents the mean of observations on 3 mice. (Values obtained in sham-operated control animals subtracted).

*Tumourigenic capacity*

In order to see whether the tumour cells of the subrenal grafts had retained their malignant character, their ability to form colonies in soft agar and to form tumours in athymic, nude mice was examined. Single cell suspensions of the grafts,

**Figure 8** Infiltration of subrenal xenograft by mouse cells. Sections taken from the rim of a human osteogenic sarcoma, TPX. *Left panel*, no pretreatment,; *right panel*, host animals pretreated with $200\,mg\,kg^{-1}$ of Cy on the day before implantation of the graft. (H & E × 156).

taken on Day 6 gave rise to colonies in 7/9 cases studied (not shown).

When the grafts were removed from the kidneys and implanted s.c. in athymic, nude hosts (5 grafts for each tumour tested), out of the 12 different tumours studied altogether 10 grew subcutaneously in the new hosts (6 malignant melanomas, 2 colon carcinomas and 2 sarcomas).

In some instances we had occasion to measure the growth rates of the grafts after re-implantation in athymic mice. It was found that the tumours grew subcutaneously with tumour volume doubling times that were not statistically different from those observed before passage under the renal capsule ($P<0.6$), indicating that the tumours had retained their characteristic growth rates (data not shown).

### Discussion

The use of human tumour xenografts in chemotherapy studies is based on the premise that the transplanted human tumour cells retain important properties, including their chemosensitivity, when growing in the foreign host. Although it can not be excluded that some selection occurs when human tumours are transplanted into athymic, nude mice, there is considerable evidence that upon subsequent serial transplantation they maintain their properties and that they generally reflect rather well the chemosensitivity of the parent tumours (Sordat *et al.*, 1974; Povlsen & Jacobsen, 1975; Houghton & Taylor, 1978; Nowak *et al.*, 1978; Shorthouse *et al.*, 1980; Giuliani *et al.*, 1981; Steel *et al.*, 1983). For this reason s.c. growth of human tumours in athymic mice has been used as a reference system for other assays of chemosensitivity (Tveit *et al.*, 1982; Aamdal *et al.*, 1983*a*, 1984*b*). However, evidence that human tumour xenografts transplanted under the renal capsule of immunocompetent mice retain their properties had not been presented.

It was found here, in agreement with our previous findings (Aamdal *et al.*, 1984*a*, 1984*b*, 1984*c*), that a variety of xenografts from different human tumour lines was capable of growing well under the renal capsule of conventional mice, supporting the view that the conditions under the renal capsule of mice are favourable for growth of human tumours (Bogden *et al.*, 1979).

Several lines of evidence indicated that the subrenal grafts retain morphological and functional characteristics during the assay period of 6 days. Thus, after 6 days under the renal capsule the tumour tissue resembled closely the xenograft of origin. Proliferating tumour tissue with numerous mitoses and little or no necrosis was found. In the instances where characteristic substances could be demonstrated, the tumour tissue retained the ability to produce these substances after growth under the renal capsule.

Of particular significance is our demonstration that the grafted tumour cells had retained their malignant growth potential after 6 days under the kidney capsule. This follows from the demonstration that they were able to form colonies in soft agar and s.c. tumours in athymic mice. The tumours formed had growth rates that were closely similar to those of the original xenografts in athymic mice, indicating that the subrenal tumours present after 6 days were representative of the original s.c. xenografts and were not derived from a selected subpopulation of the tumour cells.

Our results differ from those of Edelstein *et al.* (1983) and Seltzer *et al.* (1983) who reported that subrenal human grafts consisted mainly of oedema, inflammatory cells, granulation tissue and necrotic tissue. Part of the discrepancy may be due to the fact that we have used serially transplanted and fairly rapidly growing tumours whereas these authors largely used surgical specimens directly from patients. However, this can hardly account for the whole difference as Edelstein *et al.* (1983) studied xenografted tissue as well. Obviously, it is essential that only viable tissue is grafted. In our hands the results improved markedly with experience over a period of several years.

The assumption that the cell-mediated responses to a heterograft does not become evident until after the 6-day observation period (Bogden *et al.*, 1979) has not been confirmed (Edelstein *et al.*, 1983; Aamdal *et al.*, 1984*b*). The present results show that an immune response may appear already after a few days. Thus, antibodies directed against the transplanted human tissue could be detected already on Day 3, and the infiltration of the graft by host cells was seen already on Day 4.

The infiltration of the subrenal grafts by host cells is a potential source of error in the measurement of the growth of the tumour tissue. However, in a separate study (Aamdal *et al.*, 1984*b*) where the contribution of the mouse cells to the total graft volume was quantified, it was found that in these tumour lines, the mouse cell infiltration did not significantly affect the growth of the grafts during the 6-day observation period.

The present study is part of an extensive investigation of the 6-day SRC assay and its merits compared to other procedures used for studying the response of human cancer cells to cytostatic drugs. In other papers we have reported that the chemosensitivity of several human tumour xenografts was closely similar when tested in the 6-day SRC and in the athymic, nude mouse model (Aamdal *et al.*,

1983*a*, 1984*c*, 1984*d*). Altogether these results, which recently have been confirmed in more extensive studies (to be published), provide evidence that human tumour lines transplanted under the kidney capsule of conventional mice may be a useful system for screening of new chemotherapeutic drugs. Further studies on specimens obtained directly from patients are required to establish the validity of the 6-day SRC assay as a method for predicting the chemosensitivity of individual human tumours.

The authors are indebted to Dr A. Godal for carrying out the titration of anti-human antibody and to Dr T. Rognum, Department of Pathology, The National Hospital, Oslo, for carrying out the cytochemical demonstration of CEA. We wish to thank Ruth Holm, M.Sc., for help with the immunohistochemical staining. The excellent technical assistance of Unni Rønning and Marianne Isaksen is gratefully acknowledged. S. Aamdal is a Fellow of the Norwegian Cancer Society.

### References

AAMDAL, S., FODSTAD, Ø., JOHANNESSEN, J.V. & PIHL, A. (1983*a*). Chemosensitivity testing *in vivo*. Comparison of the subrenal capsular assay in immunocompetent mice with the nude mouse model. *Proc. 13th Internatl Congress of Chemotherapy, part 227*, Vienna, p. 22.

AAMDAL, S., FODSTAD, Ø., TVEIT, K.M., JOHANNESSEN, J.V. & PIHL, A. (1984*a*). Validity of the subrenal capsular assay in conventional mice. *Int. J. Cell Cloning*, **1**, 287.

AAMDAL, S., FODSTAD, Ø. & PIHL, A. (1984*b*). Human tumor xenografts transplanted under the renal capsule of conventional mice. Growth rates and host immune response. *Int. J. Cancer*, **34**, 725.

AAMDAL, S., FODSTAD, Ø. & PIHL, A. (1984*c*). The six-day subrenal capsule assay for testing the response of human tumors to anti-cancer agents. Validity in cancer research and treatment. *Ann. Chir. Gynaecol.*, (in press).

AAMDAL, S., FODSTAD, Ø. & PIHL, A. (1984*d*). Reduced anti-neoplastic activity of *cis*-dichloro-diamine-platinum(II) administered with high salt concentration in the vehicle. Studies in mice. *J. Natl Cancer Inst.*, **73**, 743.

BELLET, R.E., DANNA, V., MASTRANGELO, M.L. & BERD, D. (1979). Evaluation of a "nude" mouse-human tumor panel as a predictive secondary screen for chemotherapeutic agents. *J. Natl Cancer Inst.*, **63**, 1185.

BOGDEN, A.E., KELTON, D.E., COBB, W.B. & ESBER, H.J. (1978). A rapid screening method for testing chemotherapeutic agents against human tumor xenografts. In: *Proceedings of the Symposium on the Use of Athymic (Nude) Mice in Cancer Research*. (Eds. Houchens & Ovejera), New York: Gustav Fischer, p. 231.

BOGDEN, A.E., HASKELL, P.M., LEPAGE, D.J., KELTON, D.E., COBB, W.R. & ESBER, H.J. (1979). Growth of human tumor xenografts implanted under the renal capsule of normal mice. *Exp. Cell. Biol.*, **47**, 281.

BOGDEN, A.E., COBB, W.B., LEPAGE, D.J. & 5 others. (1981). Chemotherapy responsiveness of human tumors as first transplant generation xenografts in the normal mouse. *Cancer*, **48**, 10.

COBB, W.R., BOGDEN, A.E., REICH, S.D., GRIFFIN, T.W., KELTON, D.E. & LEPAGE, D.J. (1983). Activity of two phase I drugs, homoharringtonine and tricyclic nucleotide, against surgical explants of human tumors in 6-day subrenal capsule assay. *Cancer Treat. Rep.*, **67**, 173.

EDELSTEIN, M.B., FIEBIG, H.H., SMINK, T., VAN PUTTEN, L.M. & SCHUCHHARDT, C. (1983). Comparison between macroscopic and microscopic evaluation of tumour responsiveness using the subrenal capsule assay. *Eur. J. Cancer Clin. Oncol.*, **19**, 995.

FODSTAD, Ø., OLSNES, S. & PIHL, A. (1977). Inhibitory effect of abrin and ricin on the growth of transplantable murine tumors and of abrin on human cancers in nude mice. *Cancer Res.*, **37**, 4559.

FODSTAD, Ø., AASS, N. & PIHL, A. (1980). Assessment of tumour growth and of response to chemotherapy of human melanomas in athymic, nude mice. *Br. J. Cancer*, **41**, (Suppl. IV), 146.

GIOVANELLA, B.C., STEHLIN, J.S. & WILLIAMS, L.J. (1974). Heterotransplantation of human malignant tumors in "nude" thymusless mice II. Malignant tumors induced by injection of cell cultures derived from human solid tumours. *J. Natl Cancer Inst.*, **52**, 921.

GIOVANELLA, B.C., STEHLIN, J.S., SHEPARD, R.C. & WILLIAMS, L.J. (1983). Correlation between response to chemotherapy of human tumors in patients and in nude mice. *Cancer*, **52**, 1146.

GIULIANI, F.C., ZIRVI, K.A., KAPLAN, N.O. & GOLDIN, A. (1981). Chemotherapy of human colorectal tumor xenografts in athymic mice with clinically active drugs: 5-fluorouracile and 1-3-bis-(-2-chloroethyl)-1-nitrosourea (BCNU). Comparison with doxorubicin derivatives: 4′deoxydoxorubicin and 4′-0-methyldoxorubicin. *Int. J. Cancer*, **27**, 5.

GODAL, A., FODSTAD, Ø. & PIHL, A. (1983). Antibody formation against the cytotoxic proteins abrin and ricin in humans and mice. *Int. J. Cancer*, **32**, 515.

GRIFFIN, T.W., BOGDEN, A.E., REICH, S.D. & 6 others. (1983). Initial clinical trials of the subrenal capsule assay as a predictor of tumor response to chemotherapy. *Cancer*, **52**, 2185.

HOUGHTON, J.A. & TAYLOR, D.M. (1978). Maintenance of biological and biochemical characteristics of human colorectal tumours during serial passage in immune-deprived mice. *Br. J. Cancer,* **37,** 199.

KOPPER, L. & STEEL, G.G. (1975). The therapeutic response of three human tumor lines maintained in immune-suppressed mice. *Cancer Res.,* **36,** 2704.

MEERA KAHN, P. (1971). Enzyme electrophoresis on cellulose acetate gel: Zymogram patterns in man-mouse and man-Chinese hamster somatic cell hybrids. *Arch. Biochem. Biophys.,* **145,** 470.

McDOWELL, E.M. & TRUMP, B.F. (1976). Histologic fixatives suitable for diagnostic light and electron microscopy. *Arch. Pathol. Lab. Med.,* **100,** 405.

NOWAK, K., PECKHAM, M.J. & STEEL, G.G. (1978). Variation in response of xenografts of colo-rectal carcinoma to chemotherapy. *Br. J. Cancer,* **37,** 576.

OSIEKA, R., HOUCHENS, D.P., GOLDIN, A. & JOHNSON, R.K. (1977). Chemotherapy of human colon cancer xenografts in athymic nude mice. *Cancer,* **40,** 2640.

OVEJERA, A.A., HOUCHENS, D.P. & BARKER, A.D. (1978). Chemotherapy of human tumor xenografts in genetically athymic mice. *Ann. Clin. Lab. Sci.,* **8,** 50.

POVLSEN, C.O. & JACOBSEN, G.K. (1975). Chemotherapy of a human malignant melanoma transplanted in the nude mouse. *Cancer Res.,* **35,** 2790.

ROGNUM, T.O., BRANDTZAEG, T., OERJASAETER, H., ELGJO, K. & HOGNESTAD, J. (1980). Immunohistochemical study of secretory component, secretory IgA and carcinoembryonic antigen in large bowel carcinomas. *Pathol. Res. Pract.,* **170,** 1101.

SELTZER, S., McCORMICK, K.J., PANJE, W.R., PLATZ, C.E. & MERROCK, R.H. (1983). Assessment of the subrenal capsule (SRC) assay in the determination of chemotherapeutic sensitivities of head and neck cancers. *Proc. Am. Assoc. Cancer Res.,* **1261,** 319.

SHORTHOUSE, A.J., SMYTH, J.F., STEEL, G.G., ELLISON, M., MILLS, J. & PECKHAM, M.J. (1980). The human tumour xenograft – A valid model in experimental chemotherapy? *Br. J. Surg.,* **76,** 715.

SORDAT, B., FRITSCHÉ, R., MACH, J.-P., CARRE, L., OZZELLO, L. & CEROTTINI, J.-C. (1974). Morphological and functional evaluation of human solid tumours serially transplanted in nude mice. *Proc. First International Workshop on Nude Mice,* Stuttgart: Gustav Fischer Verlag, p. 269.

STEEL, G.G., COURTENAY, V.D. & PECKHAM, M.J. (1983). The response to chemotherapy of a variety of human tumour xenografts. *Br. J. Cancer,* **47,** 001.

TVEIT, K.M., FODSTAD, Ø., LOTSBERG, J., VAAGE, S. & PIHL, A. (1982). Colony growth and chemosensitivity *in vitro* of human melanoma biopsies. Relationship to clinical parameters. *Int. J. Cancer,* **29,** 533.

TVEIT, K.M. & PIHL, A. (1981). Do cell lines *in vitro* reflect the properties of the tumours of origin? A study of lines derived from human melanoma xenografts. *Br. J. Cancer,* **44,** 755.

VENDITTI, J.M. (1981). Preclinical drug development: Rationale and methods. *Semin. Oncol.,* **8,** 349.

WATSON, R.D., SMITH, A.G. & LEVY, J.G. (1975). The detection by immunodiffusion of tumour associated antigenic components in extracts of human bronchogenic carcinoma. *Br. J. Cancer,* **32,** 300.